# Novel Sensitizing Agent Formulation for Bulk Emulsion Explosives with Improved Energetic Parameters

**DOI:** 10.3390/ma15030900

**Published:** 2022-01-25

**Authors:** Bartlomiej Kramarczyk, Mateusz Pytlik, Piotr Mertuszka, Katarzyna Jaszcz, Tomasz Jarosz

**Affiliations:** 1 NITROERG S.A., 1 Alfred Nobel Square, 43-150 Bierun, Poland; b.kramarczyk@nitroerg.pl; 2 Department of Physical Chemistry and Technology of Polymers, Silesian University of Technology, 44-100 Gliwice, Poland; katarzyna.jaszcz@polsl.pl; 3 Conformity Assessment Body, Central Mining Institute, 1 Gwarków Square, 40-166 Katowice, Poland; mpytlik@gig.eu; 4 KGHM CUPRUM Ltd. Research & Development Centre, 2-8 Sikorskiego Street, 53-659 Wrocław, Poland; piotr.mertuszka@kghmcuprum.com

**Keywords:** emulsion explosive, velocity of detonation, ability to perform mechanical work

## Abstract

Bulk emulsion explosives, although they are very convenient and safe to use, also have disadvantages, with the main one being the relatively low power in relation to cartridged emulsion explosives or classic nitroesters (e.g., dynamites). Therefore, materials of this type currently have only limited use. In addition, these materials are characterized by the variability of blasting parameters over time from loading into the blasthole, which is closely dependent on the utilised mining method of the mine, which makes it difficult to precisely control the fragmentation. The industry is trying to respond to the demand for bulk emulsion explosives with increased energy and improved parameter stability, but so far it has not been possible to do so in a safe and effective way. Methods of improving blasting parameters mainly rely on additives to oxidant solutions during production, which creates additional risks at the production stage, as it involves handling hot and concentrated ammonium nitrate solutions, for which there are known cases of uncontrolled decomposition of such solutions, even leading to an explosion. This paper presents a method of improving the thermodynamic parameters and the stability of the sensitization reaction without the need for changes in the oxidant solution.

## 1. Introduction

Emulsion explosives (EEs) are a fairly recently developed and constantly evolving class of energetic materials [1,2,3] that feature high safety parameters and excellent performance benchmarks, comparable in some cases to the performance of dynamites. EEs are obtained by sensitizing an EE matrix—a water-in-oil emulsion of ammonium nitrate—either physically (e.g., with the use of glass microspheres) or chemically (e.g., through tractions, in which gas is released within the entire volume of the matrix), with the two types of processes being used to produce cartridged and bulk EEs, respectively. Regardless of their form of use, the development of EEs with continuously improving properties is a highly-active and multidisciplinary field that attracts significant scientific interest, focused on various aspects of those materials, be it their rheology [4,5], their energetic properties [6,7] or their safety features [8]. Most of the recent developments in the field focus on introducing a variety of additives into the EE formulations [9,10].

In the case of bulk EEs, which are produced in-situ using special mixing and loading units, the possibility of supplementing the EE with powdered raw materials is very limited. This results from the fact that static mixers, which are widely used in mixing-pumping units, are not suited to mixing solid components, with any larger solid aggregates posing the risk of blocking the mixer, making further loading impossible. Consequently, the sensitizing agent must be employed in the form of a liquid, so as to provide lubrication of the loading hose.

In the case of cartridged EEs, the above limitations can be avoided and it is therefore much easier to modify the detonation parameters of such blasting agents. Many studies report the use of demilitarised high explosives, such as TNT [11], RDX [12], Composition B, HMX and NC/NG-based smokeless powders [13], as supplements to EEs, allowing disposal of those explosives and increasing the energetic parameters of such supplemented EEs formulations [14], but those high explosives need not originate from reclamation efforts, as pristine high explosives are reported to be used as EE sensitizing supplements [15]. It should be noted, however, that emulsion explosives should, by design, consist of safe and non-explosive components, supplementing them with high explosives does not comply with this design and compromises the increased production, handling and usage safety of emulsion explosives.

Among other potential additives to EEs, the use of concentrated hydrogen peroxide was also investigated, as a possible method of minimising the amount of harmful gases (i.e. carbon monoxide, nitrogen oxides) emitted upon detonation [16,17]. The drawback to this approach is that concentrated hydrogen peroxide is highly reactive, with even traces of contaminants or elevated temperatures inducing its decomposition. Consequently, alternative methods of sensitizing EEs, without compromising their safety features, are of significant research interest. Titanium and magnesium hydrides are examples of some among the newly proposed sensitizing agents [18,19]. Although such supplements are theoretically promising, they are not without drawbacks. To exemplify, in the case of metal hydrides, which are highly reactive, the risk of hydrogen evolution and formation of an explosive atmosphere needs to be considered (particularly so in conditions of poor ventilation, such as is common in underground mining). Additionally, the price and sophisticated storage requirements result in the use of metal hydrides as EE supplements being impractical.

Additions of perchlorates to the solutions of the oxidising phase of emulsions are also known [20], but due to the production process, where we are dealing with concentrated solutions of ammonium nitrate at a temperature of about 90 °C, adding any reactive ingredients may increase the risk of uncontrolled decomposition or even explosion. It has been proven that aluminium additives do not give satisfactory results [21]. Additionally, the presence of aluminium can cause problems during production because it requires the use of additional dosing systems. Blasting fumes containing Al_2_O_3_ have a negative effect on the health of employees. All of the above methods of improving parameters may pose a safety risk. The main assumption of emulsion explosives is to achieve a high degree of safety during production, transport, use and decontamination of waste. Therefore, including high-energy additives directly in the emulsion matrix should be avoided, as it should remain a safe system of primary oxidising agents and safe fuels. The sensitizer should not be a hazardous substance, as well. High concentrations of sodium nitrite increase the risk of poisoning. In the case of contamination, nitrogen oxides may be released. Such a formulation of components allows them to remain classified into transport category 5.1 (oxidising agent). The assumption of the work was not to change the composition of the emulsion matrix. The change in the formulation of the sensitizer was aimed at improving the sensitization parameters (speed and stability) with an additional improvement of detonation parameters and obtaining a more favorable and ecological composition of blasting fumes.

The current reference composition of the sensitizing component is comprised by approx. 4.5 wt.% of active ingredients and as much as 95.5 wt.% of water. Introducing water into the mixed bulk EEs reduces its sensitivity to detonating stimuli, increases the critical diameter and negatively affects the detonation parameters, lowering the velocity of detonation and the explosion temperature. The presented research topic aims to develop safe bulk EEs that exhibit increased energy. The most promising of the considered solutions seems to be the replacement of a part of the water of the sensitizing component with oxidizing, reducing and active sensitizing components. The mixture contained in the new component during detonation would react in an explosive way, increasing the energy effect of the reaction, while active sensitization is to support the gas sensitization process, the optimal form of which is nitrogen gasification.

Sensitizing the EE charges chemically results in the formation of gas bubbles in the bulk of the charge. This process is a type of foaming and its progression is associated with a gradual decrease in the density of the charge over time. The rate of the reaction, through which gas is formed is dependent on temperature. This dependence is undesirable, particularly when conducting blasting operations in rocks, whose temperature is either particularly high or particularly low, as it can significantly accelerate or decelerate the progression of the EE sensitizing process. Unfortunately, within safe pH values of the oxidising solution in the EE matrix, the sensitization rate is strictly dependent on the temperature of the components. Typically, the EE charges become sensitive to initiation once their density drops below 1.25 g/cm^3^, although the degree of mixing of the components is also a key factor that needs to be taken into account. The performance of the charges is strongly dependent on their density [22,23]. The instability of density over time is a significant issue that limits the application of chemically sensitized bulk emulsion explosives. The key aims of this work were to:

- Improve the detonation parameters of new bulk explosives,
- Improve the quality of the spoil fraction,
- Increase the sensitivity of bulk explosives to shock and reduce their critical diameter,
- Improve the stability of bulk EE performance over time,
- Streamline and improve the reliability of existing loading systems in the context of their application in underground and open pit mines,
- Increase the oxygen balance value and limit the amount of nitrogen oxides and carbon monoxide produced during blasting,
- Maintain the current transport classification (Class 5.1, as per the guidelines for the transport of dangerous goods [24]) and the level of safety during production and transport.

## 2. Materials and Methods

### 2.1. Materials

#### 2.1.1. Components of the Emulsion Explosive Formulation

All tests were performed on the same batch of emulsion matrix (supplied by Nitroerg S.A.) for underground bulk emulsion formula, containing ammonium nitrate, calcium nitrate, water, oil, emulsifier and auxiliary components.

The main sensitizing component was an aqueous solution of sodium nitrite (>95%, Standard Sp. z o.o., Lublin, Poland). This was used to sensitize the standard product Emulinit 8L bulk emulsion explosive matrix. As a modification, sensitizing components were made in which, in addition to sodium nitrite as a sensitizing agent, auxiliary components such as ammonium nitrate (fertilizer grade, Zakłady Azotowe Puławy, Puławy, Poland), sodium perchlorate (>95%, Arkema, Colombes, France), pH modifier and dye were added. The inclusion of these components is what differentiates the BK-1 and BK-2 formulations from the previous Emulinit 8L bulk emulsion explosive formulation. The relevant details of the composition of the EE matrix and tested EE formulations are given in Table 1.

#### 2.1.2. Auxiliary and Reference Materials

1,3,5-Trinitro-1,3,5-triazinane (RDX) and flaked 2,4,6-trinitrotoluene provided by Nitrochem (Bydgoszcz, Poland) were used as reference explosive materials for investigating the ability of the samples to perform mechanical work and for investigating brisance via the Hess method.

The probes used for velocity of detonation (VoD) measurements were produced by MREL, model PROBEROD (electrical resistivity 331.7 Ω/m) were used. The lead rods (purity > 99.97%, Φ = 40 mm) were used as received.

### 2.2. Experimental Procedures

#### 2.2.1. Preparation of Emulsion Explosives and Experimental Samples

The explosive components were blended mechanically in a plastic vessel with a capacity of 2 dm^3^ using an electrically powered mechanical stirrer equipped with a propeller-shaped stirring rod. The components were stabilized to a constant temperature of 25 degrees Celsius and the mixing took place each time at the same temperature. The material prepared in this way was quickly elaborated into charges and conditioned at a constant temperature for a period of 3 h at constant temperature. The large batches of each explosive type were used as the reservoir, from which samples for the individual tests were taken.

Samples for microscopic observations were placed on the base microscope glass slide in the form of thin layers.

Samples for the determination of the composition of post-detonation gases were prepared in glass pipes, with one end sealed with clay, due to the fact that glass and clay are inert materials that are known not to interfere with gas analysis.

The samples for the ballistic mortar test were produced by mixing the components by hand, due to the sample amounts being insufficient for using the mechanical stirrer. After mixing, 10 g samples were accurately weighed.

The fundamental properties and performance parameters of the tested EE formulations were predicted using EXPLO5 software provided by OZM Research s.r.o. and the most relevant parameters were verified experimentally, as described in the following subsections.

#### 2.2.2. Investigation of the Density of Charges over Time

Open vessels with a set volume of 115 cm^3^ were weighed and loaded with the EE samples without leaving any voids or bubbles. Any excess of the EE was scraped off from the top of the cup to maintain the set volume of the samples, followed by weighing of the vessel. This was repeated every 5 min for the first hour, as well as after 180 and 1440 min have elapsed, in order to establish a time-resolved density profile for each of the investigated EE formulations.

#### 2.2.3. Microscopic Observations

A ZEISS Primotech polarised light microscope was used to observe the structure of samples. Both the pure EE matrix and complete EE formulations (Emulinit 8L, BK-1, BK-2) were observed, at a magnification of 10×.

#### 2.2.4. Determination of the Ability to Perform Mechanical Work

The ability of the EE charges to perform mechanical work was evaluated using a ballistic mortar. The detonation of a set mass (10 g charges were used, as is most common) of an explosive propels the mortar, moving it out of equilibrium. The maximum angle, to which the mortar was moved out of equilibrium, is recorded and used as the measure of the ability of the sample to perform mechanical work. Due to the nature of this method, it is most suitable for comparative trials. Therefore, a reference explosive is employed, against which the samples are tested. In this work, 1,3,5-trinitro-1,3,5-triazinane (RDX) was used as the reference material, with flaked 2,4,6-trinitrotoluene acting as a secondary reference material. Consequently, the relative ability of a sample to perform mechanical work (*X*) is given as a relative value (% of the ability of RDX to perform mechanical work), calculated using Equation (1).

(1) $$X=\frac{m}{m_{R}}\cdot 100\%$$

where:

- *m*—arithmetic average of (1−cos(α)) values for the tested explosive
- *m_R_*—arithmetic average of (1−cos(α)) values for the reference explosive

#### 2.2.5. Determination of Air Blast Parameters

**Figure 1:**
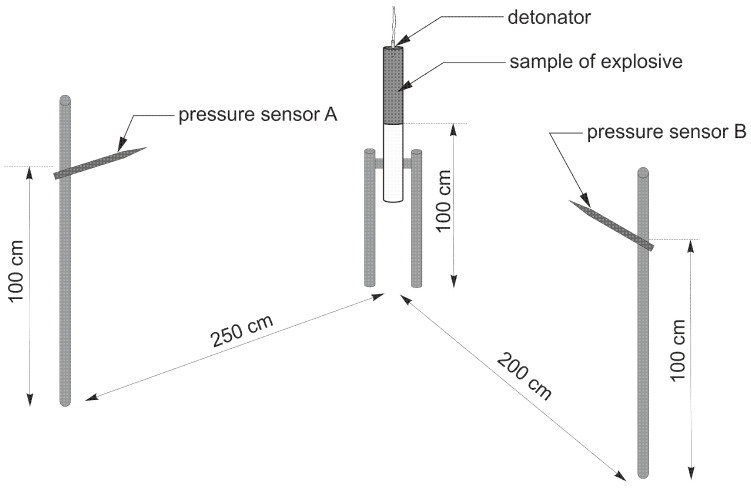
Outline of the experimental set-up for determining shock wave parameters.

The parameters of the shock wave generated by the investigated EE charges were determined using two pressure sensors—type 137B23B piezoelectric pressure sensors (PCB PIEZOTRONICS) were used. In this test, the EE charge was hung vertically, 100 cm above the ground. The pressure sensors were placed at different angles, at a distance of 200 and 250 cm, respectively, from the axis of the tested EE samples (Figure 1). Sensor data was recorded using a DEWESoft SIRIUS high-speed amplifier coupled with a computer capable of sampling data at a frequency of 1 MHz.

#### 2.2.6. Determination of Detonation Velocity

**Figure 2:**
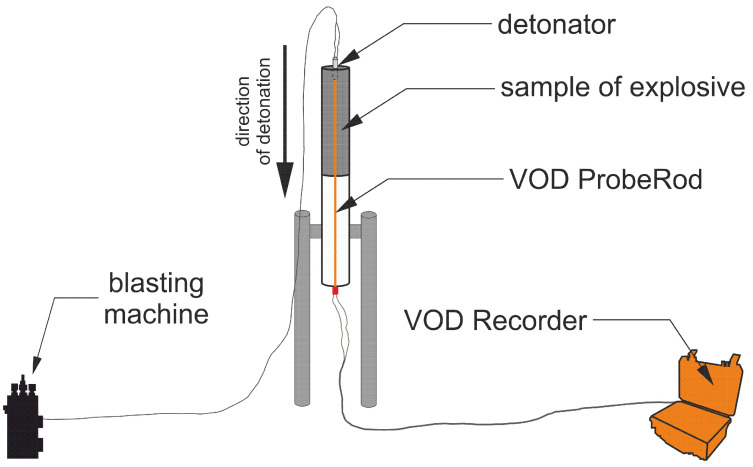
Schematic depiction of the experimental set-up for measuring velocity of detonation.

**Figure A1:**
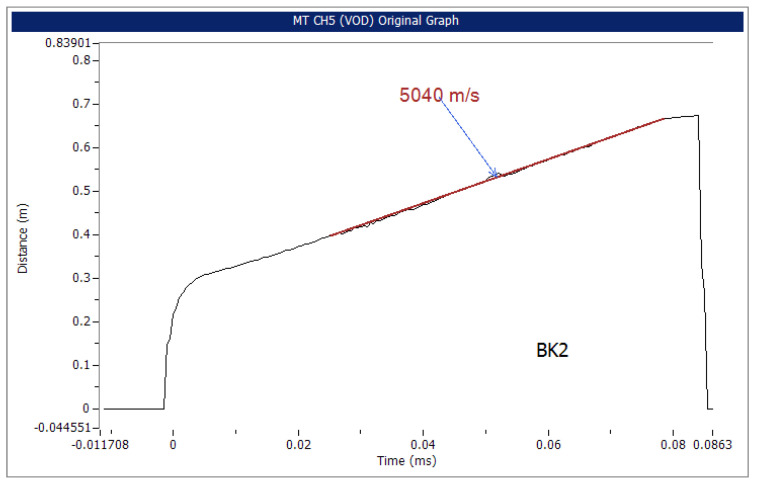
Sample distance vs. time curve, from which the velocity of detonation for the BK2 EE formulation was determined graphically.

Detonation velocity values were investigated by the electrical method, based on the change of electrical resistance of a conducting probe, using a MicroTrap (MREL, Kingston, ON, Canada) velocity of detonation recorder. The VOD ProbeRod with a unit resistance of 331.7 ohm/m was inserted axially into the EE sample from the opposite end of the detonator. Then the ProbeRod was connected to the coaxial cable to transmit the signal to a MicroTrap™ VOD/Data Recorder, as shown in Figure 2. The detonation velocity experiments were conducted after the sensitised charges were left to stand in ambient conditions for 3 h. Samples were prepared by filling clear glass tubes, length of 500 mm, internal diameter of 46.4 mm and wall thickness of 1.8 mm, with the explosive. The VoD value was determined from the slope of the distance vs. time curve recorded for the VoD probe, as exemplified by the sample curve presented in Figure A1.

#### 2.2.7. Determination of the Composition of Post-Detonation Gases

**Figure 3:**
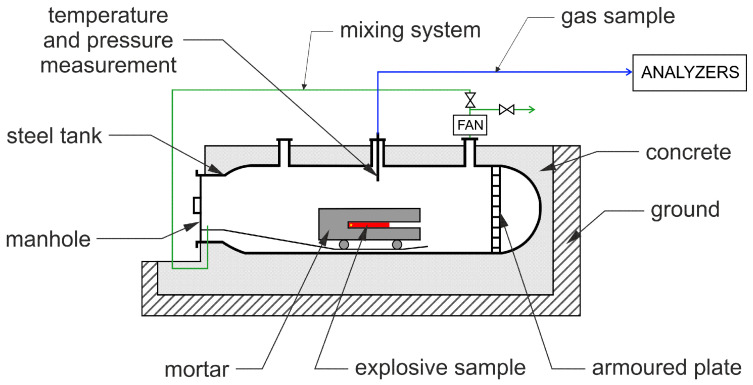
Blasting chamber for post-detonation gases analysis (based on [29]).

The experiments were conducted in accordance with the EN 13631-16:2006 standard [29]. The EE charges of a set mass of 530 g were placed in glass tubes and stemmed with clay. The load was placed in the mortar inside the blasting chamber with a volume of 15 m^3^ (Figure 3). The tested explosive is initiated from the bottom by a secondary charge of 650 mg pentaerythritol tetranitrate. After the detonation of the tested charge, a mixing system is run for three minutes, in order to homogenize the gas mixture composition in the entire volume of the test chamber. The amounts of toxic oxides in the post-detonation gases were determined using continuous measurement chemiluminescent (TOPAZE 32M using a dual chamber for NO, NO_X_ and NO_2_ determination) and infrared (MIR 25 for CO and CO_2_ determination) absorption analyzers. The concentration of each gas is recorded 20 min after the detonation of the test charge. Based on these values, the volume of each gas generated per unit mass (1 kg) of the explosive is calculated. The tests are conducted for three samples of each tested explosive and the final result is the average of the three values.

Interpretation of the results of the analysis is conducted based on the fact that the concentrations of carbon monoxide (CO) and carbon dioxide (CO_2_) is constant after the initial mixing period (homogenization of the post-detonation gas mixture). Due to the occurrence of consecutive reactions between the detonation products, the initial concentrations of nitrogen oxides (NO, NO_X_ and NO_2_) are determined by plotting a dependence of the concentration of each substance over time that has elapsed since detonation and extrapolating the experimental curve to the moment of detonation [30]. The initial concentrations of post-detonation gases (*C_G_*) are used to calculate the quantity of each gas (*Q_G_*) at normal conditions (273 K, 760 mm_Hg_) via Equation (2):

(2) $$Q_{G}=\frac{V_{\mathrm{ch}}\cdot 273}{10^{6}\cdot m\cdot 760}\;\cdot \frac{p_{1}}{T_{1}}\;\cdot C_{G}\;\left[\frac{{\mathrm{dm}}^{3}}{\mathrm{kg}}\right]$$

where:

- *p_1_*—measured pressure in the chamber after detonation [mm_Hg_]
- *T_1_*—measured temperature in the chamber after detonation [K]
- *V*_ch_—volume of the experimental chamber [dm^3^]
- *m*—mass of the detonated explosive sample [kg]

#### 2.2.8. Determination of Brisance via the Hess Method

**Figure 4:**
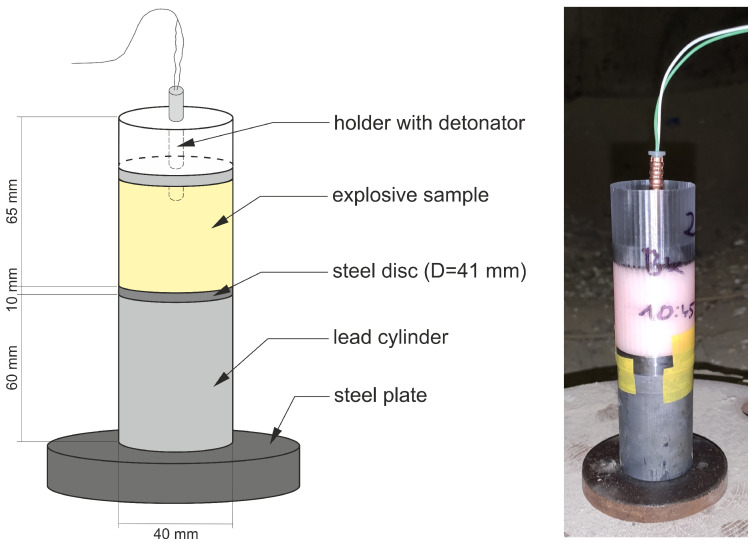
Experimental setup for determining brisance using the Hess method.

A lead cylinder (99.97% purity, diameter 40±0.2 mm, height 60±0.15 mm; face surfaces were machined to 10 grade) was placed vertically on the ground. A cylindrical steel disc (1.7035 steel, diameter 41±0.2 mm, height 10±0.2 mm; face surfaces were machined to 2.5 grade and hardened to 150–200 HB) was placed on top of this cylinder. A set mass (50 g) of the tested sample, loaded into a 3D printed plastic (PET-G) testing cup (inner diameter 40 mm, height 65 mm), was placed onto this plate and initiated, as depicted in Figure 4. This resulted in axial compression of the lead cylinder, with the change in the height of this cylinder being used as a measure of brisance. Similarly to other procedure for the ballistic mortar test, RDX was used as the reference material. The sample was initiated using a standard 0.65 g PETN detonator.

## 3. Results

### 3.1. Projected Detonation Parameters

The properties of the EEs utilising the proposed sensitizing agent formulations were predicted theoretically in comparison with the commonly used Emulinit 8L EE, using EXPLO5 software. Although this software is an accurate tool for predicting the fundamental properties and performance parameters of a variety of explosives, based on their composition, it is unable to take into account some physical processes taking place in emulsion explosives, which are non-ideal explosives, such as the precipitation of microscopic grains of ammonium perchlorate in the bulk of the EE.

Despite the above, the software was able to predict that both BK-1 and BK-2 will outperform Emulinit 8L in terms of both velocity of detonation (VoD) and compression energy (Table 2). It is worth noting that the volume of evolving gases for BK-2 is the lowest out of the three EE formulations, despite it showing both the highest detonation pressure and VoD, indicative that the energy contained within this formulation is used significantly more efficiently in its case than in the case of the other two formulations.

### 3.2. Changes in Sample Density

The EE samples were sensitised chemically, through a reaction between ammonium nitrate(V) and sodium nitrite(III) that resulted in the gradual evolution of nitrogen gas [31]. In the case of bulk explosives, loaded directly into boreholes, control over the course of this reaction is virtually non-existent after the EE matrix and sensitizing agent are mixed. Simultaneously, however, the progress of this reaction results in a gradual decrease in EE density, affecting its detonation parameters, which is a significant issue in the planning of blasting operations. Consequently, bulk EEs, which show only a weak dependence of density on time elapsed after loading the EE into boreholes, or whose density quickly stabilises at a quasi-constant level, are highly desirable. The specific time period, in which this density stabilisation should take place, is dependent on the particular application. In the case of underground mining, 30 min are often given as the minimal time elapsed between loading boreholes with bulk EE and carrying out the blasting operation. Hence, this time period was used as a benchmark of comparison between the investigated EE formulations.

**Figure 5:**
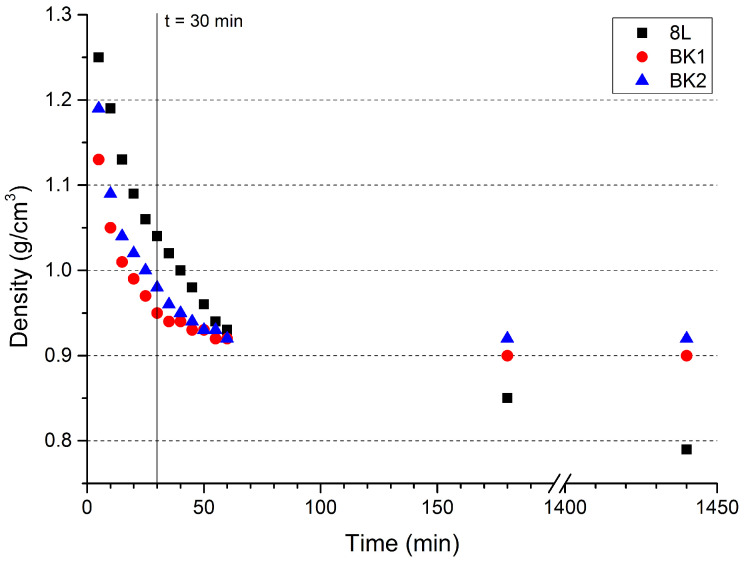
Comparison of changes in the density of the EE samples over time.

In the case of the investigated samples, the “traditional” 8L EE formulation shows an almost linear dependence of density on time elapsed since the sensitizing process began (Figure 5). Although the rate of density changes decreases slightly after approx. 20 min, there is no evidence for the density of the samples stabilising in the investigated time period and the density continues to change rapidly after the benchmark 30 min time period.

The two new formulations, BK-1 and BK-2, initially show a much faster density decrease than what is observed for 8L. Unlike 8L, the density of BK-1 and BK-2 formulations quickly begins stabilising, with density changes after 30 min have elapsed being only minor, particularly in the case of BK-1.

The postulated density stabilisation for BK-1 and BK-2 is supported by the fact that the same density values (0.90 and 0.92 g/cm^3^, respectively) are observed after 180 and 1440 min have elapsed, unlike with the 8L sample, whose density continues to decrease (0.85 and 0.79 g/cm^3^, respectively, after 180 and 1440 min have elapsed).

### 3.3. Microscopic Observations

**Figure 6:**
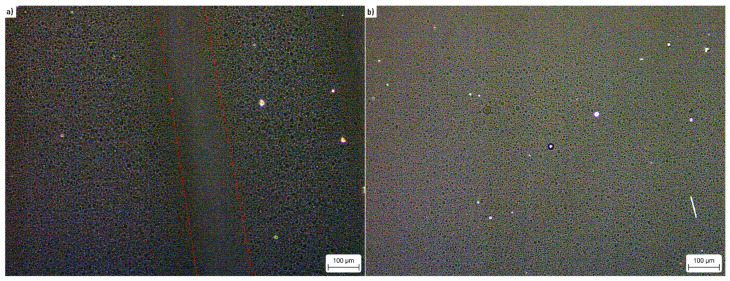
Polarised light micrograph of (**a**) Emulinit 8L; (**b**) Bulk EE matrix. The diagonal red lines indicate the region, in which sensitizing is on-going.

The EE matrix is largely amorphous (Figure 6), with only marginal amounts of crystalline species, originating from crystallisation of the EE matrix on impurities or defects. A similar amount of crystallites is present for Emulinit 8L, with the centre of the micrograph showing a vertically-aligned region with an altered structure, indicating the on-going formation of gas bubbles due to the reaction between the matrix and sensitizing agent used.

**Figure 7:**
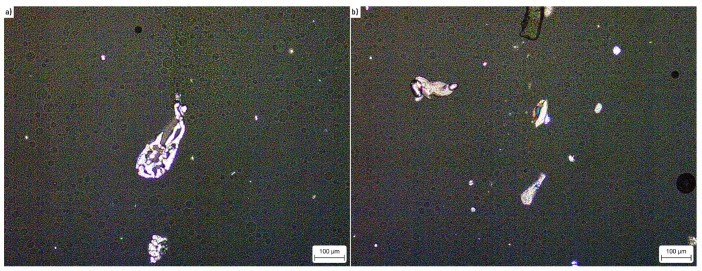
Polarised light micrograph of (**a**) BK-1; (**b**) BK-2.

In the case of BK-1 and BK-2 (Figure 7), larger crystalline species are present. These species are most likely crystals of ammonium perchlorate that precipitated in the reaction of the highly soluble sodium perchlorate with the concentrated ammonium nitrate solution. Because ammonium perchlorate is an explosive in itself, when the EEs are exposed to a shock wave, its crystals may promote the formation of additional hot spots. This phenomenon will act in synergy with the evolution of gas bubbles in the EE matrix, further sensitizing the EE sample and facilitating the evolution, propagation and maintaining the detonation wave in the EE.

### 3.4. Ability to Perform Mechanical Work

In the case of the mechanical work ability test, slightly better results were obtained for BK-1 and BK-2 compositions than for the reference material (Emulinit 8L) due to their higher energy (Table 3). This is due to the fact that there is less water in the composition in favor of the components reacting in an explosive way. A better conversion degree of reaction at a higher detonation velocity gives a higher detonation pressure, which has an impact on the projectile launch capacity.

### 3.5. Shock Wave Parameters

Very slight variations in the pressure of the blast wave were observed (Table 4). This may be due to a small difference in the detonation temperature. There are no additives, e.g., aluminium, which could increase the explosion temperature and extend the time of impact of the blast wave pressure.

### 3.6. Detonation Velocity

The lower water content in the new formulations causes the velocity of detonation (VoD) to increase (Table 5). Additionally, there is some perchlorates content which improves the propagation of the detonation wave.

### 3.7. Composition of Post-Detonation Gases

Due to the better distribution of hot spots and a certain content of ammonium perchlorate microcrystals, compositions BK-1 and BK-2 are characterized by better detonation and better conversion of reagents. Especially in the case of the BK-1 composition, the conversion rate causes the fuel oxidation reaction to trend towards the formation of carbon dioxide and water. As a result, the amount of harmful gases is partially reduced (Table 6).

### 3.8. Investigation of Brisance

**Figure 8:**
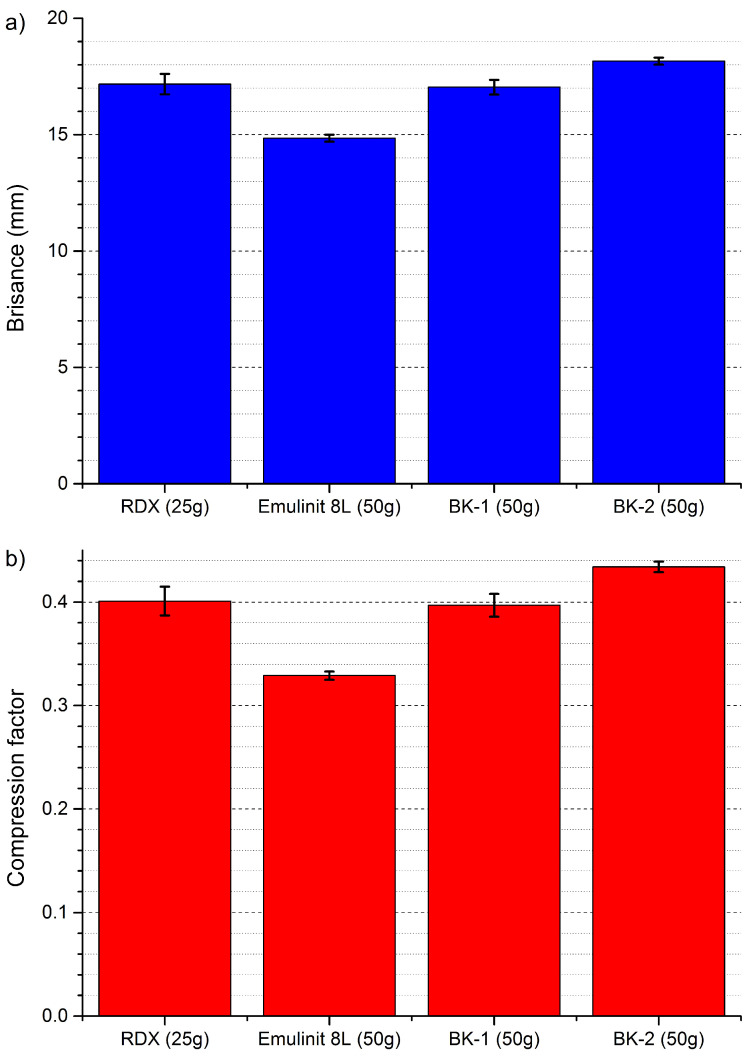
Comparison of (**a**) brisance and (**b**) compression factors determined for the tested EE samples and reference explosive (RDX) using the Hess test.

Brisance is directly dependent on the velocity of detonation. It is a measure of the effect of pressure on objects closest to the explosive. It is especially visible in the case of the BK-2 composition, because in a similar density range the detonation velocity is much higher than for the reference material (Figure 8).

## 4. Discussion

The results of the research showed that the parameters of the new BK-1 and BK-2 formulas were better in every aspect compared to the standard, commercially used Emulinit 8L emulsion explosive. In addition, the performance of BK-1 and BK-2 exceeded theoretical predictions collated with the use of EXPLO5 software. This is likely due to the precipitation of ammonium perchlorate crystals (Figure 7) in the bulk of the BK-1 and BK-2 EEs that were not present in the case of Emulinit 8L (Figure 6). These crystals are hypothesised to act as additional “hot spots”, further sensitizing the explosives and facilitating their detonation.

The inclusion of sodium perchlorate in the EE formulations (Table 1) is an interesting matter in terms of material safety. Supplementing the EE matrix with perchlorates would be a source of risk due to its processing at elevated temperatures [20]. Hence, we have opted to include sodium perchlorate in the sensitising agent formulation, as it consists primarily of water and even with the inclusion of sodium perchlorate, it remains classified as an oxidising solution. Mixing the EE matrix with the sensitizing agent formulation, upon loading the EE formulation into boreholes, results in the in situ precipitation of ammonium perchlorate. Even though this process is expected to increase the sensitivity of BK-1 and BK-2 formulations to initiating stimuli, it does not translate into any significant increase of risk. This is due to the fact that once the EE is loaded into boreholes, it is protected from virtually all sources of accidental initiation.

The sensitization of the tested formulations is much faster and more stable, so the temperature of the components does not have such a significant influence on the reaction rate. In standard conditions of underground mines (at temperatures in the range of 25–35 °C), it is capable of detonation after 5 min and the final density is obtained after approx. 30 min, while the standard reference material in these conditions is capable of reliable detonation after 30 min at the very least and achieves its final density only after more than 12 h. This aspect is very important in practice, because the temperature conditions in underground mines can vary significantly between different locations. Simultaneously, procedures employed in blasting may involve various time intervals between loading the boreholes and initiation of the explosive charges. Consequently, an explosive, whose performance changes only marginally with time or environment temperature, is both much more predictable and desirable for use than one, whose performance varies significantly.

In terms of the relative ability to perform mechanical work (Table 3), the BK-1 and BK-2 formulations achieved noticeably higher performance than Emulinit 8L and were even comparable to the performance of TNT.

Despite the current research focus on supplementing EEs with a variety of additives, the efficacy of such supplementation has its limitations, particularly since the additives are introduced only in limited amounts. This is illustrated well by considering the blast wave parameters that would be expected for the three types of EE formulation samples. The blast wave parameters are strongly tied to the detonation temperature and volume of gases evolving in the decomposition of a unit mass of the EE formulation. These parameters, in turn, are determined to a much greater extent by the chemical composition of the EE formulation rather than by its physical state. Consequently, even if the composition of the sensitizing phase, which constitutes less than 5 wt. % of the formulation, is changed significantly, as has been implemented for BK-1 and BK-2, in comparison with Emulinit 8L, the composition of the entire formulation is changed only to a small extent. Consequently, it is expected that those parameters will be similar for all three EE formulations, even though a very slight elevation of those parameters was observed for BK-1 and BK-2 in comparison with Emulinit 8L (Table 4).

Conversely, the brisance and velocity of detonation (VoD) are influenced to a much greater degree by physical factors than blast wave parameters. In this aspect, the introduction of a modified sensitizing phase yields the most outstanding improvement, with the brisance of BK-1 and BK-2 increasing by 20.67% and 31.91%, respectively, in comparison with Emulinit 8L (Figure 8). Simultaneously, the VoD was observed as 9.8% and 18.9% greater for BK-1 and BK-2, respectively, than for Emulinit 8L.

In the case of the BK-1 and BK-2 formulations, blasting fumes have a lower content of toxic gases compared to the standard formula. This is due to a better conversion rate and more perfect reaction during detonation. Summarizing the results new formulas of explosives are characterized by a greater efficiency of the explosive transformation, a more controlled and faster sensitization reaction and a more favorable composition of blasting fumes. The first tests under operating conditions were also carried out. The resulting explosive is capable of working properly. The obtained results require confirmation in subsequent tests carried out under operating conditions, the aim of which will be to improve the performance of the formulations via optimising the process of mixing components, which should confirm the results and conclusions obtained in laboratory conditions. In fact, the price of the new explosive bulk emulsion will be higher, but it is expected to allow carrying out blasting works in a wider range of conditions. The increased brisance of the two formulations is also expected to facilitate the use of EEs in the exploitation of hard rocks.

## 5. Conclusions

Based on the presented experimental results, we can conclude that the modified sensitising agent formulations are an all-round improvement in comparison to the traditional formulation used for Emulinit 8L. Although the performance of BK-1 was slightly inferior to that of BK-2, the sensitizing agent formulation contains approx. 20 wt. % more water than the one used for BK-2 (Table 1). This translates directly to BK-1 having a lesser unit cost than BK-2, while affording better performance than Emulinit 8L. Consequently, we envision BK-1 to serve as the general replacement for Emulinit 8L, while BK-2 may be used for more specialised applications and with some further optimisation will have the potential to serve as a replacement for nitroester-based explosives (e.g., dynamites) due to its high relative ability to perform mechanical work (Table 3), velocity of detonation (Table 5) and brisance (Figure 8).

The optimisation of the two formulations can also be targeted at fine-tuning the sensitizing agent composition to further shorten the sensitising time of the explosives and to further promote their ability to perform mechanical work. Regardless of optimisation pathway, both types of the BK EE formulations are promising energetic materials with broad future applications, possibly setting a new direction for development in the field of modern emulsion explosives.

## Figures and Tables

**Table 1:** Summary of the components of the tested explosive formulations.

EE Matrix			
**Component**	**Concentration (wt.%)**		
Ammonium nitrate	55–60		
Calcium nitrate	15–20		
Organic phase	5–7		
Water	12–15		
**Tested sensitizing agent formulations** ^1^			
**Component**	**Concentration (wt.%)**		
	**Emulinit 8L**	**BK-1**	**BK-2**
Ammonium nitrate	-	30	47
Water	95.45	61.6	41
Sodium perchlorate	-	4	8
Sodium nitrite	4.5	3	3.3
pH modifier and dye	0.05	1.5	0.7

^1^ The Emulinit 8L formulation has been described in earlier works [21,25,26,27,28].

**Table 2:** Summary of detonation parameters calculated using EXPLO5 software.

Parameter/Sample	Emulinit 8L	BK-1	BK-2
Density (g/cm^3^)	0.85	0.90	0.92
Oxygen balance (%)	0.129	0.356	0.485
**Detonation parameters at the C-J point**			
Heat of detonation (kJ/kg)	2865	2931	2987
Detonation pressure (GPa)	4.40	4.98	5.24
Velocity of detonation (m/s)	4400	4594	4678
Volume of evolved gases (dm^3^/kg)	992	987	983
Compression energy (kJ/kg)	691	725	741

**Table 3:** Summary of the ability of the tested explosive charges to perform mechanical work.

Explosive	Mortar Inclination Angle [deg]	Relative Ability to Perform Work	Ref.
RDX (reference explosive)	17.20	100%	-
Dynamite	-	84%	[32]
ANFO	-	51%	[32]
TNT (flaked)	14.13	67.7%	-
Emulinit 8L	13.60	62.6%	-
BK-1	14.23	68.8%	-
BK-2	14.33	69.6%	-

**Table 4:** Summary of the results of air blast parameter tests. Each value given is the of at least three independent measurements.

Explosive	P_MAX_ [kPa] ^a^		Duration [ms] ^b^		Impulse [Pa · s] ^c^	
	Ps_2m_ ^d^	Ps_2.5m_	Ps_2m_	Ps_2.5m_	Ps_2m_	Ps_2.5m_
Emulinit 8L	123.73±7.91	73.33±7.02	1.27±0.01	1.46±0.02	57.20±0.89	43.83±0.60
BK-1	127.13±5.67	74.30±1.01	1.26±0.01	1.49±0.04	57.53±0.86	44.37±0.67
BK-2	131.20±6.49	75.93±1.71	1.27±0.01	1.46±0.03	58.60±1.04	44.80±0.26

^a^ Peak overpressure recorded during the experiment; ^b^ Air blast positive phase duration; ^c^ Air blast total positive phase impulse; ^d^ Position of the sensor, with the subscript value denoting the distance of the sensor from the axis of the EE sample.

**Table 5:** Summary of the results of velocity of detonation measurements.

Detonation Velocity [m/s]				
Explosive	Sample 1	Sample 2	Sample 3	Average
Emulinit 8L	4220	4270	4210	4233±32
BK-1	4650	4650	4650	4647±6
BK-2	5040	5030	5030	5033±6

**Table 6:** Summary of the average composition of post-detonation gases for the tested explosives.

Emulinit 8L	CO_2_	CO	NO_2_	NO
Concentration [ppm]	4583 ± 45	162 ± 11	1.4 ± 0.2	20.0 ± 7.4
Unit mass emission [dm^3^/kg]	114.8 ± 1.1	4.11 ± 0.28	0.04 ± 0.01	0.51 ± 0.19
**BK-1**	**CO_2_**	**CO**	**NO_2_**	**NO**
Concentration [ppm]	4664 ± 6	100 ± 4	1.5 ± 0.2	11.6 ± 2.8
Unit mass emission [dm^3^/kg]	117.1 ± 0.9	2.51 ± 0.12	0.04 ± 0.01	0.29 ± 0.07
**BK-2**	**CO_2_**	**CO**	**NO_2_**	**NO**
Concentration [ppm]	4553 ± 24	136 ± 18	1.2 ± 0.2	11.0 ± 5.3
Unit mass emission [dm^3^/kg]	115.3 ± 0.4	3.45 ± 0.46	0.03 ± 0.01	0.28 ± 0.13

## Data Availability

Data is available on request from any of the Authors.
